# Phytochemical fingerprint and biological activity of raw and heat-treated *Ornithogalum umbellatum*

**DOI:** 10.1038/s41598-023-41057-w

**Published:** 2023-08-23

**Authors:** Aytül Uzun Akgeyik, Emine Yalçın, Kültiğin Çavuşoğlu

**Affiliations:** 1 Science and Technology Application and Research Center, Yozgat Bozok University, Yozgat, Turkey; 2 Department of Biology, Faculty of Science and Art, Giresun University, Giresun, Turkey

**Keywords:** Biotechnology, Molecular biology

## Abstract

The plants that we use as food in our daily diet and as risk preventers against many diseases have many biological and pharmacological activities. The heat treatments applied during the cooking of the plants cause changes in the phytochemical content and bioactivity. In this study, the phytochemical fingerprint and biological activities of raw and heat-treated extracts of *Ornithogalum umbellatum* L., which is widely consumed in the Black Sea region, were investigated. The bulb and leaf parts of the plant consumed as food were dried in an oven at 35 °C and then ground into powder. For heat treatment, the plant was boiled at 100 °C for 20 min. Differences in phytochemical contents of raw and heat-treated extracts were determined by ICP-MS, LC–MS/MS, and FTIR analysis. Biological activity was investigated with antiradical, antimicrobial, antimutagenic and antiproliferative activity tests. In this way, the effect of heat treatment on both the phytochemical content and biological activity of the *O. umbellatum* extract was determined. Gallic acid, procateuic acid and caffeic acid were found as the main compounds in the *O. umbellatum* extract, while the presence of procateuic aldehyde, vanillin and kaempferol in minor proportions was determined. There was a significant decrease in phenolic compound levels after heat treatment and gallic acid content decreased by 92.6%, procateuic acid content by 90% and caffeic acid content by 84.8%. Significant differences were detected in macro and micro element levels after heat treatment in ICP-MS results. While Cd, Ba and Zn levels of the raw extract increased; Na, Mg, K, Fe, U, Co levels decreased significantly. In FTIR spectrum, shifts and disappearances were observed in some of the vibrations and the emergence of new vibrations was also determined after heat treatment. Raw extract exhibited strong scavenging activity against H_2_O_2_ and DPPH and had a broad spectrum antimicrobial property. As a result of heat application, regressions were detected in antiradicalic, antibacterial and antifungal activities. Antimutagenic and antiproliferative activities were determined by the *Allium* test and a significant decrease in both activities and loss of activity against some chromosomal abnormalities were determined after heat treatment. While the antiproliferative activity of the raw extract was 20%, the activity of the heat-treated extract decreased to 7.6%. The raw extract showed the strongest antimutagenic effect with 69.8% against the unequal distribution of chromatin. Similarly, the antimutagenic activity of the extract, which reduced the bridges by 56.1%, decreased to 0.74% after heat treatment and almost lost its antimutagenic activity. The biological activities of raw *O. umbellatum* are closely related to the major compounds it contains, and the decrease in the levels of these compounds with the effect of heat was reflected in the activity. Studies investigating the phytochemical contents of plants are very important and the studies investigating biological activities related to phytochemical content are more remarkable. In this study, the phytochemical fingerprint of *O. umbellatum* was determined, its biological activities were related to the compounds it contained, and the biological activity was found to be heat sensitive.

## Introduction

Many plant species used as food are consumed after applying various cooking methods. Heat treatment of herbal foods causes physical, biological and textural changes. As a result of the inhibition of microorganisms by the cooking process, food safety, digestibility and bioavailability increase. In addition to the beneficial effects of cooking, it can also have harmful effects such as decreased nutritional quality, loss of nutrients, formation of undesirable compounds such as acrylamide, loss of texture and color^1^. In many studies, it has been reported that cooking has different effects on the chemical composition of plants. Especially the heat treatment applied during cooking causes serious changes in the structure of phytochemicals. These changes vary according to the composition of the active ingredients of the plant, the molecular structure of the phytochemical, the type and duration of the heat treatment^2^. Especially the decrease in the amount of phenolic compounds and the formation of intermediate products as a result of breakage in chemical bonds are among these changes. Lower molecular weight polyphenols can be formed as a result of breaking the chemical bonds of high molecular weight polyphenols. In some cases, the phenolic compounds in the herbal extract can also be converted to each other by the effect of heat^3^. It has been reported in the literature that heat treatment applications cause a decrease in phenolic content and accordingly a decrease in the antioxidant capacity and radical scavenging properties of the plant^4^. In some studies, it has been reported that an increase in antioxidant activity is observed with the effect of new phenolic compounds formed after heat treatment. These differences observed in the literature studies can be explained by the changes in the new compounds produced by the action of heat and by the activity of these compounds^3^.

In this study, the effect of heat application on the phytochemical content and biological functions of *O. umbellatum*, which is consumed as a food in the Black Sea region, especially in Giresun (Turkiye), was investigated. *Ornithogalum* (Hyacinthaceae, Ornithogaloideae) has more than 200 species and can grow in different climatic conditions. In the world and Turkiye, parts of *O. umbellatum* such as leaves, stems and roots are consumed as food. *Ornithogalum* species have many biological activities and these effects are closely related to the active ingredients they contain. The phytochemical content of *O. umbellatum* species includes phenolic compounds such as gallic acid, cinnamic acid, chlorogenic acid, and flavonoids such as quercetin, rutin, epicatechin. The phytochemical content of *O. umbellatum* species also varies according to the environment in which it spreads and the stress conditions it is exposed. These differences in phytochemical content are due to changes in secondary metabolites produced by *Ornithogalum* species during their adaptation to their environment. Many biological activities of plants result from the action of secondary metabolites^5,6^. A diet rich in fruits and vegetables containing secondary metabolites reduces the risk of different chronic diseases such as cancer and diabetes. Disease risk-reducing effects of secondary metabolites in humans occur through complementary and pleiotropic mechanisms such as regulation of the detoxification system, activation of the immune system, antiangiogenic, antimutagenic, antioxidant and antibacterial effects. Many plants rich in secondary metabolites are consumed both as food and added to the daily diet for protection against many diseases^2,7^. There are many studies in the literature investigating the biological functions and phytochemical contents of herbal extracts. The rich plant diversity in the world and Turkiye makes these studies insufficient. The secondary metabolites produced by a plant species under different climatic conditions and stress vary, resulting in plant samples of the same species collected in different regions differing in phytochemical and biological activity^8^. In studies based on the phytochemical analysis carried out with plants consumed as food, the raw form of the plant is generally used. Many plant consumed in the diet are subjected to cooking processes and the applied heat treatment causes a change in the phytochemical content. This change is also reflected in biological activity. In the literature, there are some studies on the phytochemical analysis and antioxidant activities of *O. umbellatum* samples collected from different regions^9,10^. However, there is no study investigating the effect of heat application during cooking on the phytochemistry and activity of *O. umbellatum*. In this study, considering these deficiencies in the literature, the phytochemical contents and various biological activities of raw and heat-treated extracts of *O. umbellatum* were investigated. *O. umbellatum* is consumed by roasting, frying and mostly boiling in the Black Sea region. Therefore, in this study, the bulbs and aerial parts of the *O. umbellatum*, which parts are consumed as food, were boiled and subjected to heat treatment and compared with the raw samples. After the heat treatment application, the changes in the biological activity together with the changes in the chemical content were also evaluated. Phytochemical analysis of raw and heat-treated *O. umbellatum* extracts was carried out by investigating macro and micro element levels with ICP-MS, by examining biochemical functional groups in macromolecules with FTIR, and by detecting phenolic compounds in LC/MS–MS. As a result of these analyses, the phytochemical fingerprint of *O. umbellatum* was created using advanced chemical techniques and the changes in the chemical composition after heat treatment were determined. In the second stage of the study, the biological activities of raw and heat-treated extracts were investigated. For this purpose, antiradical, antibacterial, antifungal, antimutagenic and antiproliferative activities of the extracts were investigated and the effects of heat treatment on these activities were also determined.

## Material and methods

The chemicals used in all analyses in the study were of high purity and were obtained from Sigma-Aldrich and Merck, and carmine (CAS: 1390-65-4) was obtained from Isolab. *Allium cepa* L. (2n = 16) bulbs used as bioindicators in antimutagenic and antiproliferative activity analyses were obtained from Akdeniz Agriculture (TR-55-K-009228).

### Sample collection and extraction

**Figure 1:**
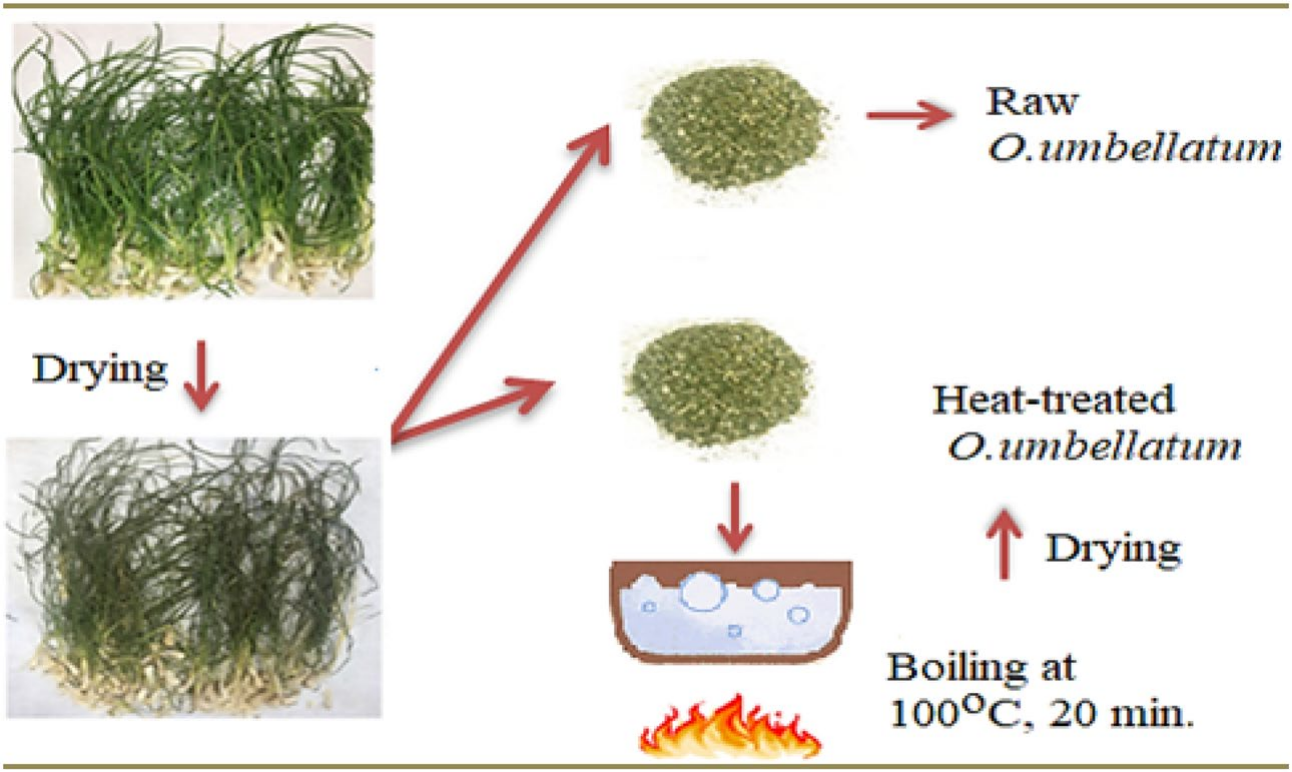
Preparation of raw and heat-treated *O. umbellatum*.

*O. umbellatum*, which is consumed as food in the Black Sea region, was collected from the Kumbet highland (Giresun, 40.560136, 38.436516) by random sampling in November 2019 and the species identification was made in the Department of Botany of Giresun University according to the “Flora of Turkey”^11^. A specimen of the plant was archived in the herbarium with the voucher number BIO-Orum221/2019. Experimental research and field studies on plants, including the collection of plant material, comply with relevant institutional, national, and international guidelines and legislation. In the study, the bulb and leaf parts of the plant consumed as food were used in phytochemical analysis and biological activity studies. The diseased and wilted parts of the plant were separated and the healthy parts of the plant were dried in an oven at 35 °C and then ground into powder. No treatment was applied to some of the powdered samples and they were named as raw samples. Some of the powdered samples were boiled in water at 100 °C (1:5) for 20 min, dried in an oven and called as heat-treated samples (Fig. 1).

Extraction of raw and heat-treated samples was carried out by maceration using three different solvents: methanol, water and chloroform. 2 g of sample was extracted in 100 mL solvent at room temperature for 24 h and then filtered with filter paper (Whatman No:4). After centrifugation for 10 min at 10,000 rpm, the supernatant was evaporated with an evaporator (Heidolph, Hei-VAP ML). The residues obtained after the evaporation of the solvent were stored in glass bottles at + 4 °C^12^. Extract efficiency in extraction with each solvent was determined by using the following Eq. (1).

1 $$ {\text{Extract yield }}\left( \% \right){:}\;{\text{ Extract obtained }}\left( {\text{g}} \right){\text{/Amount of plant }}\left( {\text{g}} \right) \times {1}00 $$

The total amount of phenolic substances was also taken into account in determining the extraction efficiency. The total amount of phenolic substance was determined by the Folin-Ciocaltaeu method^13^. 2.5 mL of Folin-Ciocaltaeu reagent and 7.5 mL of sodium carbonate solution were added to 0.5 mL of extract from each solvent. The solutions were incubated for 2 h at room temperature. The same procedures were repeated with gallic acid at different concentrations. At the end of the period, the absorbance of all solutions was measured spectrophotometrically at a wavelength of 750 nm, and the total amount of phenolic substance was expressed as mg gallic acid equivalent (GAE)/g extract.

### Quantitative phytochemical analyses

Phytochemical analysis of raw and heat-treated *O. umbellatum* samples was performed by LC–MS/MS, FTIR and ICP-MS analysis. With the data obtained from these analyses, the fingerprints of the raw and heat-treated samples were determined and the effects of heat treatment on the phytochemical content were evaluated.

### LC–MS/MS analysis

Raw and heat-treated samples (2 g) were extracted with methanol:dichloromethane (4:1) solvent in an ultrasonic bath for 120 min, filtered through a 0.45 µM syringe filter and analyzed by LC–MS/MS (Thermo Scientific). 0.1% Formic acid–water (A) and methanol (B) were used as solvents. Solvent program was applied as: A: 100% for 0–22 min; A:5% for 22–25 min; A:0% for 25–30 min. Flow rate, column furnace temperature and injection volume were determined as 0.7 mL/min, 30 °C and 20 µL, respectively. ODS Hypersil 4.6 * 250 mm column was used. The MS/MS analysis conditions are as follows: capillary temperature: 300 °C, vaporizer temperature: 350 °C, sheat gas pressure: 30 Arb, Aux gas pressure: 13 Arb, positive polarity: 2500 V, negative polarity: 2500 V, discharge current: 4 µA^14,15^. Quantification was evaluated according to MS/MS. The precision, accuracy, and selectivity of the method were evaluated by repeating the measurements at three concentrations for each compound. A good precision was determined, and the method was found to show high selectivity and accuracy. LC–MS/MS analysis was carried out by Hitit University- HÜBTUAM.

### ICP-MS

Raw and heat-treated plant samples (0.1 g) were burned in a microwave solubilization (Milestone D5, USA) device for ICP-MS analysis. The burning process was carried out by adding 5 mL Suprapur nitric acid + 2 mL hydrogen sulfide on the sample and transferred to the teflon tubes of the microwave. After incineration, 50 mL ultrapure water was added to the sample and analysis was performed with the ICP-MS device (Thermo Scientific ICAP QC, USA). The analysis parameters are as follows: nebulizer gas: 0.96 L/min, radiofrequency power: 1550 W, plasma gas: 0.88 L/min, nebulizer pressure: 3.01 bar, spray chamber temperature: 3.7 °C. Calibration curves were created with the standards prepared in the range of 0.1–1500 ppb. Each measurement of samples and standards was repeated three times. ICP-MS analysis was carried out by Yozgat Bozok University-Science and Technology Application and Research Center.

### FTIR

Analyses were performed using the FTIR (Perkin Elmer Frontier FTIR NIR and FIR Spectroscopy) device. This device allows direct analysis from the solid and allows analysis without the pellet preparation step. FTIR plots of raw and heat-treated *O. umbellatum* samples were drawn with the Spectra manager program. FTIR analysis was carried out by Yozgat Bozok University-Chemistry Deparment.

### Biological activities

Biological activity analyses were performed with methanol extract, which achieved high efficiency in *O. umbellatum* extraction. The antiradical, antibacterial, antifungal, antimutagenic and antiproliferative activities of the extract were investigated. In our previous study, it was determined that the raw extract of *O.umbellatum* exhibited a strong efficacy in the dose of 20 mg/mL^5^. In this study, a dose of 20 mg/mL was preferred for antibacterial, antifungal, antimutagenic and antiproliferative activity tests.

### Antimicrobial activities of raw and heat-treated extract

The disk diffusion test was used to determine the antimicrobial activities of *O. umbellatum* extract. Antibacterial activities of the extracts against *Escherichia coli* ATCC 25922, *Pseudomonas aeruginosa* ATCC9027, *Salmonella enteritidis* ATCC13076, *Bacillus subtilis* IMG 22, *Bacillus cereus ATCC 14579, Staphylococcus aureus* ATCC 25923 were tested. Antifungal activity was investigated against *Candida tropicalis* ATCC 13803 and *Candidia albicans* ATCC 90028 species. Gram-positive and Gram-negative bacteria were incubated in Mueller Hinton Broth medium at 37 °C for 48 h and a sample of 10^8^ cells/mL (0.5 McFarland) from the culture was spread evenly on the surface of Mueller–Hinton agar petri dishes. Fungi were first incubated in Potato Dextrose Broth medium for 48 h, and 10^6^ CFU/mL (0.5 McFarland) samples taken from the culture were homogeneously inoculated on Sabouraud Maltose Agar petri dishes. Empty sterile discs with a diameter of 6 mm were placed on the media surfaces and 20 µL of extract was transferred to the discs. Petri dishes were kept at 4 °C for 1 h and then incubated for 24 h at 37 °C for bacteria and 27 °C for fungi. Antimicrobial activity was determined by measuring incubation zones (mm)^16^. Nystatin and Amikacin antibiotics were used as standard.

### Radical scavenging activities of raw and heat-treated extract

Radical scavenging activities were tested against DPPH and H_2_O_2_. DPPH scavenging activities of raw and heat-treated samples were performed according to the procedure described by Gündüz et al.^17^ 2.4 mL of DPPH was mixed with 1.6 mL of extract (50–150 µg/mL). The solution was left in the dark for 30 min, and then the absorbance was measured spectrophotometrically at 517 nm. The experiment was repeated three times at each concentration. BHT was used as a standard and DPPH radical scavenging activities of the extracts were calculated by using the following Eq. (2).

2 $$ {\text{DPPH radical scavenging activity }}\left( \% \right) \, = \, \left( {{\text{A}}_{0} - {\text{ A}}_{{1}} } \right)/{\text{A}}_{0} \times { 1}00 $$

A_0_ is the absorbance of the control and A_1_ is the absorbance of the extract or standard solution.

The H_2_O_2_ scavenging test was carried out according to the method of Ruch et al.^18^. 3.4 mL of extract and 0.6 mL of H_2_O_2_ were mixed and the absorbance of the reaction mixture was measured spectrophotometrically at 230 nm. A blank solution containing the phosphate buffer without hydrogen peroxide was used in the measurements. BHT was used as a standard and the experiment was repeated three times at each concentration. The percentage of hydrogen peroxide scavenging was determined by using the following Eq. (3).

3 $$ {\text{Scavenging of H}}_{{2}} {\text{O}}_{{2}} \left( \% \right) \, = \, \left[ {\left( {{\text{A}}_{{\text{C}}} {-}{\text{ A}}_{{\text{S}}} } \right)/{\text{A}}_{{\text{C}}} } \right] \, \times { 1}00 $$

where A_C_ is the absorbance of the control, A_S_ is the absorbance in the presence of the extract or standard.

### Antimutagenic activity

The antimutagenic activity of *O. umbellatum* extract was determined using the chromosomal abnormality (CA) test. The CA test was applied using the *Allium* test and six different groups were formed for this purpose. Group I was accepted as negative control and germinated with distilled water^19^. Group II, designated as the positive control, was treated with sodium azide (NaN_3_), a potent mutagen. To determine the potential mutagenic effect of the extract alone, Group III and Group IV were germinated with 20 mg/mL raw and heat-treated extract, respectively. In Group V and Group VI, 20 mg/mL NaN_3_ + 20 mg/mL raw and 20 mg/mL NaN_3_ + 20 mg/mL heat-treated extract were applied, respectively. The antimutagenic effects were determined by examining the reduction in chromosomal abnormalities induced by positive mutagen^8^. CA (%) and antimutagenic activity (%) were calculated by using the following Eqs. (4,5).

4 $$ {\text{CA }}\left( \% \right) \, = {\text{A}}/{\text{B }} \times {1}00 $$

5 $$ {\text{Antimutagenic activity }}\left( \% \right) \, = \, \left[ {\left( {{\text{a}} - {\text{b}}} \right)/\left( {{\text{a}} - {\text{c}}} \right)} \right] \, \times { 1}00 $$

A: refers to the cell with total abnormal chromosome, B: refers to the total counted cell, a: represents the %CA of the NaN_3_ applied group, b: represents the %CA of the plant extract + NaN_3_ applied group, c: represents the %CA of the control group.

### Antiproliferative activity

The antiproliferative effect of *O. umbellatum* extracts was determined by examining the changes in mitotic index (MI) ratios in *Allium cepa* root tip cells. MI is an indicator of cell proliferation. A decrease in the MI value below 22% in organisms compared to the control group indicates a lethal effect, while a decrease below 50% generally indicates a sublethal effect. Inhibition of the MI is associated with a delay in cell death or cell proliferation kinetics^20,21^. Four different groups were formed for the determination of antiproliferative activity. While the control group was germinated with distilled water, the positive control group was germinated with 20 mg/mL glyphosate and the application groups with 20 mg/mL raw extract and heat-treated extract. *Allium* bulbs of equal size (n = 10) in each group were germinated for 72 h in glass beakers. At the end of the germination period, root slides were prepared and proliferated cells were examined^22,23^. The antiproliferative activity was evaluated based on the decrease in cell proliferation. A total of 5.000 cells were counted for each group and MI percentages were calculated by using the following Eq. (6).

6 $$ {\text{Cell proliferation }}\left( {{\text{MI }}\% } \right) \, = {\text{ Number of dividing cells}}/{\text{total number of cells }} \times { 1}00 $$

### Preparation of MI and CA slides

In order to determine the antiproliferative and antimutagenic activities, root tip samples of each group were collected at the end of the rooting period. Root tips of 1 cm length were fixed with Clarke solution and passed through ethanol series. After 17 min of incubation at 60 °C in 1 N HCl for hydrolysis, the samples were kept in 45% acetic acid for 30 min. Finally, cells stained with acetocarmine for 24 h were examined under a advanced-research microscope^24,25^.

### Statistical analysis

Statistical analysis was performed using the “IBM SPSS Statistics 22” package program and the data obtained were given as mean ± SD (standard deviation). Statistical significance between the means was determined by Duncan's test and One-way ANOVA, and a p value of < 0.05 was considered statistically significant.

## Results and discussion

In this study, the phytochemical fingerprint of *O. umbellatum*, which is consumed as a food in the Black Sea region, was investigated by ICP-MS, LC–MS/MS and FTIR analyses, and it’s antiradical, antibacterial, antifungal, antimutagenic and antiproliferative activities were investigated. The heat treatment applied during the cooking of plants consumed as food causes important changes in the phytochemical content and accordingly the biological activity. For this reason, the same analysis were performed with the heat-treated *O. umbellatum* samples and the effect of heat on the phytochemical content and biological activity were also investigated.

### Extraction efficiency

**Figure 2:**
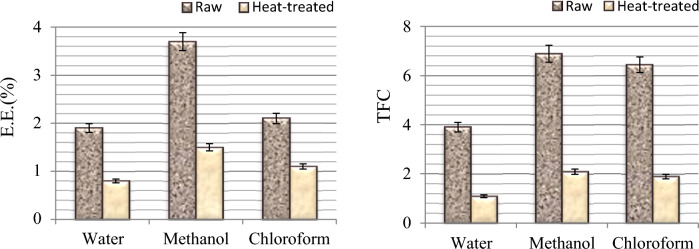
Extraction efficiency (E.E) and total phenolic content (TFC: mg GAE/g).

Raw and heat-treated plant samples were extracted by maceration method using three different solvents: water, methanol and chloroform. After extraction, extraction efficiency and total phenolic content were investigated for each solvent, and the results are given in Fig. 2. Among the solvents used, the highest extraction efficiency was obtained in methanol extraction with a rate of 3.7%. In terms of total phenolic content, the highest efficiency was obtained in methanol extraction and 6.89 mg GAE/g phenolic substance content was determined. It was determined that there were significant decreases in both extraction efficiency and total phenolic content after heat application. These reductions can be associated with the transition of many active compounds in the plant content into the boiling water. Since the efficiency and phytochemical content were determined at higher levels in methanol extraction compared to water and chloroform extraction, methanol extract was used in biological activity tests.

### LC–MS/MS analysis

**Figure 3:**
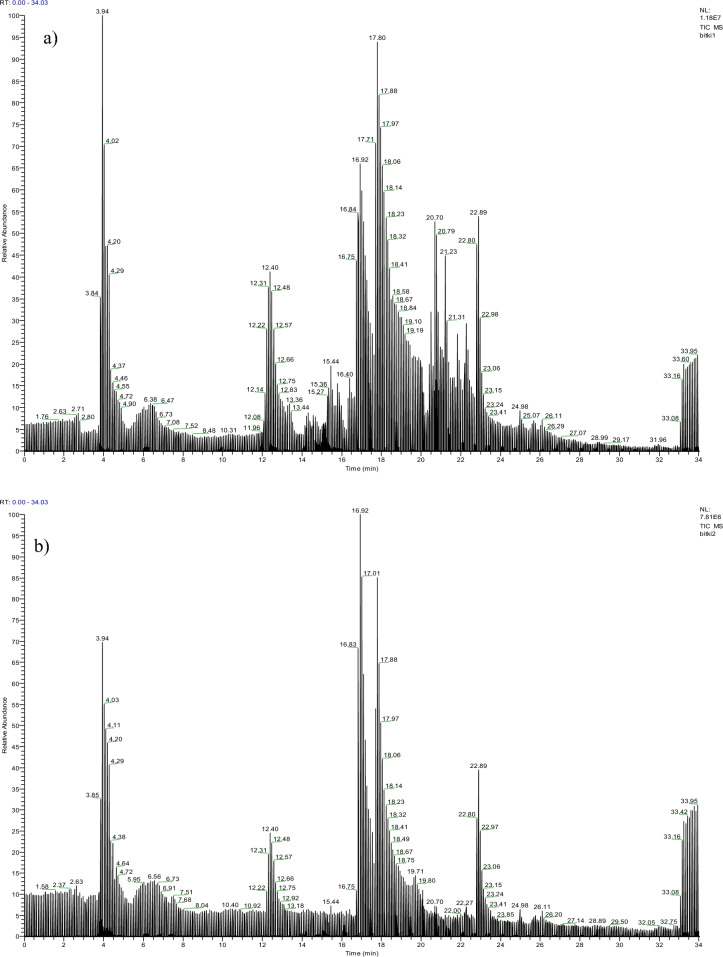
LC–MS/MS chromatograms of *O. umbellatum* extracts. Raw extract (**a**)^5^ and heat-treated (**b**) extract.

The LC–MS/MS chromatograms showing the phenolic compounds of raw and heat-treated *O. umbellatum* extracts are given in Fig. 3 and their presence rates are given in Table 1. LC–MS/MS chromatograms of all standard compounds were given in Supplementary Fig. S1. In our previous study, the presence of a total of 6 phenolic compounds was determined in the raw *O. umbellatum* extract in the analysis performed using 19 standard compounds. While gallic acid, procateuic acid and caffeic acid were found as major compounds, the presence of procateuic aldehyde, vanillin and kaempferol in minor proportions was determined in the raw extract^5^. It was determined that there was a significant decrease in the phenolic compound levels after heat treatment. Gallic acid, procateuic acid and caffeic acid were found as major compounds in the heat-treated extract similar to the raw extract, while procateuic aldehyde, epicatechin, vanillin and kaempferol were detected as minor. The heat treatment caused a 92.6% decrease in the gallic acid content, a 90% decrease in the procateuic acid content, and an 84.8% decrease in the caffeic acid content (Table 1). Similar reductions were also found in minor compounds. Unlike the raw extract, the presence of epicatechin was detected in the contents of heat-treated samples, which was associated with the formation of new compounds as a result of the degradation of existing phenolic compounds. In the literature, there are studies investigating the phytochemical content of *Ornithogalum* species. Temiz^26^ carried out the phytochemical analysis of *Ornithogalum lanceolatum* extract distributed in Mersin (Turkey) by HPLC and reported that especially the leaf parts contain intense amounts of procateuic acid, rutin, quercetin and gallic acid.

**Table 1:** Presence rates and percentage of decrease in phenolic compounds in raw and heat-treated extracts.

Phenolic substance	Amount (mg/kg)	Percentage of decrease in phenolic compounds (%)
Raw extract		
Gallic acid	41.722	
Procateuic acid	53.136	
Caffeic acid	27.162	
Procateuic aldehyde	1.305	
Vanillin	3.611	
Kaempferol	1.681	
Heat-treated extract		
Gallic acid	3.046	
Procateuic acid	5.290	
Caffeic acid	4.120	
Procateuic aldehyde	0.276	
Vanillin	0.897	
Kaempferol	0.961	
Epicatechin	0.295	

The active substances detected in the extract are responsible for the formation of many biological activities. Procateuic acid, which is detected at maximum level in raw and heat-treated extracts, exhibits a strong antioxidant activity by inhibiting free radical formation, scavenging free radicals and acting as a metal chelating agent. In addition to the antioxidant activity of procateuic acid, antimicrobial, antidiabetic, antiulcer and antiviral anti-inflammatory activities are also reported in the literature^27^. Gallic acid, which is the second major phenolic compound found in the extract and has a presence rate as 32.44% among all phenolics detected in *O. umbellatum*, is a natural herbal secondary metabolite. Gallic acid, which has many prominent pharmacological effects such as anti-inflammatory, antitumor, antioxidant, antimicrobial, antidiabetic and antiobesity, is a low molecular weight tri-phenolic compound^28^. Caffeic acid, one of the three major compounds detected in raw and heat-applied extracts, has strong biological activities. It exhibits a high antioxidant role by preventing lipid peroxidation, scavenging free radicals and increasing endogenous antioxidant enzyme activities^29^. *O. umbellatum* extract also exhibits many biological activities as a result of the cumulative effects of the phenolic substances detected in the extract content.

Heat treatment caused a decrease in the amount of phenolic compounds of *O. umbellatum*. After heat application, the amount of gallic acid, procateuic acid and caffeic acid, which are the three major compounds in the extract, decreased by 92.7%, 90%, and 84.8%, respectively. The decreases can be explained by the oxidative decomposition of phenolic compounds after heat treatment or by the formation of complexes with compounds such as tannin and anthocyanin. In some cases, the breakdown of complex phenolic compounds can also lead to the formation of new and low molecular weight structures. The detection of epicatechin after heat application, which cannot be found in the raw extract, is a good example of this situation. The decrease of phenolic content can be also related to the extraction by water while boiling, so the compounds are eliminated with enriched water. Although there are studies investigating the phytochemical content of *O. umbellatum* in the literature, there is no study investigating the effect of heat treatment on the phytochemical contents. However, changes in the content as a result of heat applications in some other plants consumed as food have been reported by many researchers. Loizzo et al.^30^ reported that the total phenolic content of 127.5 mg/g determined in *Capsicum annum* decreased to 15 mg/g after heat application. Sun et al.^31^ reported that heat treatment caused a decrease in the amount of gallic acid and chlorogenic acid in the content of *Solanum tuberosum*. In the literature, there are studies reporting that the phenolic content decreases with the effect of heat, as well as studies that found an increase in the phenolic content. Faller and Fiahlo^32^ reported that heat treatment caused an increase in phenolic content in some herbal extracts and this increase was associated with a more efficient extraction of phenolic compounds bound to the cell wall or proteins by the effect of heat.

### ICP-MS analysis

The levels of macro and micro elements detected by ICP-MS analysis in raw and heat-treated *O. umbellatum* extracts are given in Table 2. In our previous study, the content of elements in the raw *O. umbellatum* was investigated, and a high content of K was found, followed by the elements Ca, Mg, Na, Fe, and Zn. K level was determined as 1836.14 ppm, Na level as 41.95 ppm and Ca level as 486.21 ppm^5^. In the literature, it is reported that the K value of *O. umbellatum* extracts grown in different regions is between 2188.7 and 2560 mg/kg, and the Ca value is in the range of 1280–657.78 mg/kg^33,34^. Although the elements reported in the literature studies on *O. umbellatum* extract are similar, there are differences in the ratio of the elements. These differences can be associated with the phytochemical compounds produced and the biochemical reactions developed by the same species growing in different ecological environments during their adaptation to the environment. Plant foods consumed in the daily diet contain various levels of macro and micro elements. Among these, macro elements are found in high proportion compared to all other elements. The elements determined in the extract content also contribute to the healthy and organized functioning of most of the human tissues, and in this context, it is very important to get enough micro/macroelements in the daily diet. Also, the raw extract contained heavy and radioactive elements such as Co, Cu, As, Cd and U.

**Table 2:** Macro- and micro element levels of raw^5^ and heat-treated *O. umbellatum* extracts.

	Raw extract (ppm)			Heat-treated extract (ppm)		
	Mean value	SD	RSD	Mean value	SD	RSD
^23^Na	41.954	0.249	0.59	37.378	0.261	0.70
^24^Mg	167.365	1.399	0.84	160.841	0.841	0.52
^27^Al	6.910	0.113	1.63	3.160	0.037	1.16
^39^K	1836.135	18.069	0.98	1603.878	18.637	1.16
^44^Ca	486.213	8.888	1.83	387.104	6.064	1.57
^55^Mn	2.238	0.024	1.07	1.932	0.023	1.16
^57^Fe	32.780	0.131	0.40	25.556	0.357	1.40
^59^Co	0.02855	0.001	3.57	0.01945	0.000	1.18
^63^Cu	0.299	0.009	3.03	0.298	0.016	5.38
^66^Zn	7.416	0.429	5.78	7.606	0.350	4.60
^75^As	0.00419	0.001	22.68	0.00284	0.001	20.84
^88^Sr	1.509	0.016	1.08	1.481	0.056	3.77
^111^Cd	0.063	0.003	4.66	0.065	0.002	2.87
^137^Ba	2.357	0.047	1.98	2.362	0.052	2.19
^238^U	0.0024	0.000	4.68	0.00096	0.000	5.67

SD: standard deviation, RSD: relative standard deviation (%).

**Figure 4:**
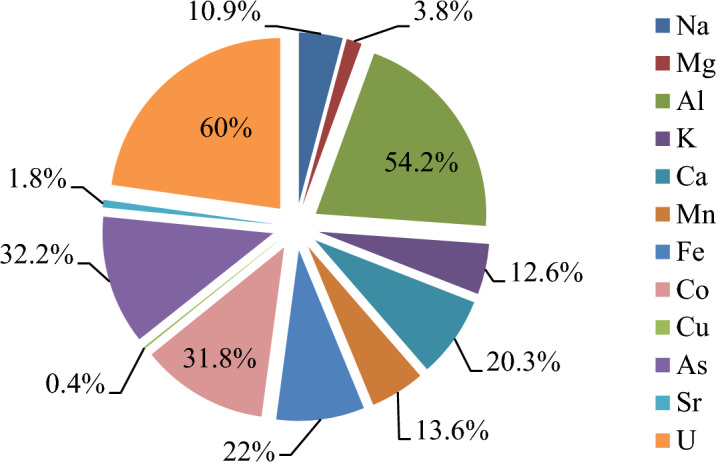
Reductions in macro- and microlement levels after heat treatment (%).

Significant differences were detected in macro and microlement levels after heat treatment. While increases were determined in Cd, Ba and Zn levels, serious decreases were detected in Na, Mg, K, Fe, U, Co, Ca levels (Fig. 4). Heat treatment resulted in a decrease in the contents of some elements; the most significant decrease was observed in element U with a rate of 60%. Similar reduction behavior was observed for anti-nutritive elements such as Al, Co and As. This result shows that the anti-nutritive elements can be purified from the plant by boiling. These decreases can be explained by the transition of the elements from the vegetative structures to the boiling water. In the heat-treated samples, it was determined that some element levels increased rather than decreased. After heat application, increases in Cd, Ba and Zn levels were determined and this increase was associated with the release of elements from cellular macromolecules with the effect of heat. In the literature, there are studies reporting that the heat treatment causes changes in element levels. Nnabugwu et al.^35^ reported important decreases in Cu, Fe, K, Ca, Mg and Na levels in raw and heat-treated *Anacardium occidentalia* samples.

### FTIR analysis

**Figure 5:**
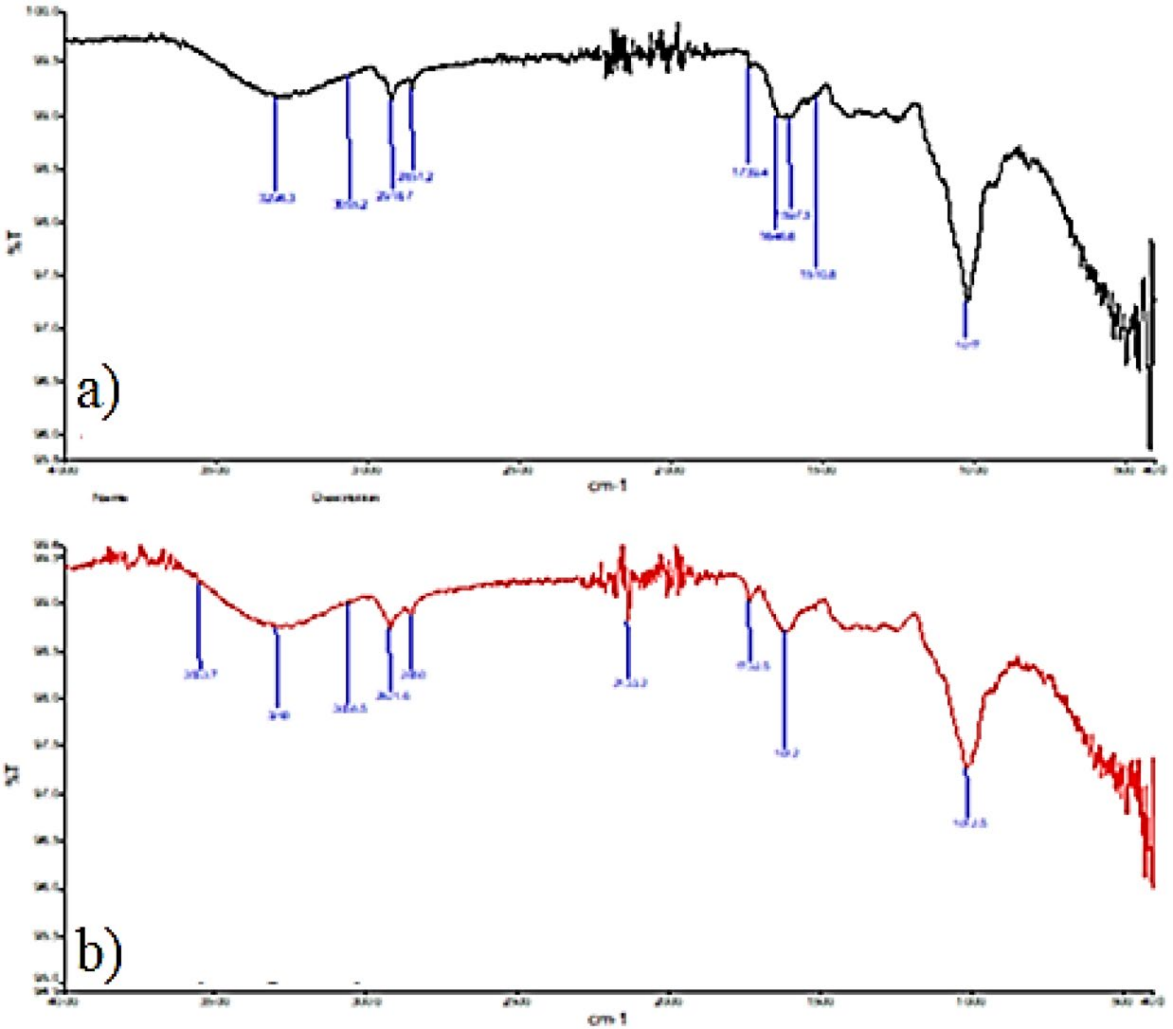
FTIR spectrum of raw *O. umbellatum* (**a**) and heat-treated *O. umbellatum* (**b**).

FTIR spectroscopy is an important analytical technique used to determine the biochemical composition of cells such as proteins, fatty acids, polysaccharides and carbohydrates. Organic compounds with –OH, –NH and –CH functional groups can be determined by FTIR spectroscopy obtained in the spectral range of 4000 to 400 cm^−1^ and changes in the composition of the samples can be evaluated. These changes are determined by considering the peak width, location and absorption intensity^36^. FTIR spectra of raw and heat-treated *O. umbellatum* samples are given in Fig. 5. In the FTIR spectrum of the raw sample, the absorption band observed at 3296.3 cm^−1^ indicates the presence of -OH and -NH groups in the sample. The same absorption band is found at 3293 cm^−1^ in the heat-treated sample. The stretching vibrations of aromatic -CH groups observed at 3055.2 cm^−1^ in the raw sample were determined at 3058.5 cm^−1^ after heat application. Absorption bands showing the aliphatic C-H tension were observed at 2918.7–2851.2 cm^−1^ in the raw sample and at 2921.6–2850 cm^−1^ in the heat-applied samples. The C=O stretching found at 1739.4 cm^−1^ was observed at 1732.5 cm^−1^ in heat-treated form. C=N peaks appearing in the 1646.6 cm^−1^ band observed in the raw sample could not be clearly observed in the heat-treated sample. The band (1646 cm^−1^) in the amide I region provides more information about the secondary structure of the proteins. Small changes were observed between band intensities in the Amid I region of raw and heat-treated samples, and these changes were associated with changes in the secondary structure of proteins with the effect of heat. This shows the nitrogen loss in the structure as a result of heat application. In the raw sample, aromatic C = C stretching at 1597.5–150.8 cm^−1^ was observed at 1612 cm^−1^ in the heat-treated sample. The C–O stress, which was in the 1017 cm^−1^ band in the raw samples, was observed at 1012.5 cm^−1^ in the treated sample. New bands were observed in the 3400–3800 cm^−1^ range in heat-treated samples, and these bands were thought to originate from the –NH tension (Amid A) and –OH groups of the proteins.

### Radical scavenging activity

**Figure 6:**
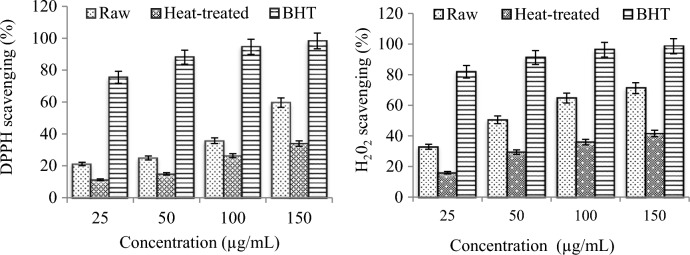
Scavenging activity of raw and heat-treated *O. umbellatum*.

The antioxidant activities of raw and heat-treated extracts were determined by radical scavenging tests and the results are given in Fig. 6. Free radical scavenging is an accepted mechanism for screening the antioxidant activity of plant extracts. The scavenging activities of the raw and heat-treated extracts increased in a dose-dependent manner. 150 µg/mL raw extract showed 59.6% DPPH removal activity and the heat-treated extract showed 33.9% activity. *O. umbellatum* extracts exhibited higher H_2_O_2_ scavenging activity compared to DPPH. At a dose of 150 µg/mL, the raw extract and the heat-treated extract exhibited 71.2% and 41.6% H_2_O_2_ scavenging activity, respectively. These results show that heat treatment application causes a decrease in free radical scavenging activity. This decrease is directly related to the change in phenolic content. Antiradical activities of phenolic compounds show a positive correlation with the number of hydroxyl groups attached to the aromatic ring. Gallic acid, which contains three hydroxyl groups, which is intensely present in the raw extract, is considered a strong DPPH and H_2_O_2_ scavenger^37^. Therefore, gallic acid contributes highly to the radical scavenging activity of *O. umbellatum*. After heat application, the gallic acid level decreased by 92.7% and this decrease was also reflected in the scavenging activity. Similarly, caffeic acid and protocatechuic acid, which have more than one hydroxyl group, also exhibit strong radical scavenging effects. Important decreases were detected in these major phenolic compounds in the extract after heat application. The radical scavenging activity of the extract also decreased as a cumulative effect of the reduction in all phenolic compounds with heat application. Although there is no study in the literature reporting the effect of heat treatment on the antioxidant activity of *O. umbellatum*, similar changes in many herbal extracts have been investigated. Jaiswal et al.^38^ reported similar reduction trends in various vegetables due to degradation and/or leaching of certain phenolic compounds responsible for DPPH activity. Jiménez-Monreal et al.^39^ reported a 27.9% loss in lipid peroxyl scavenging activity and a 74.8% loss in hydroxyl scavenging activity and no change in ABTS scavenging activity of *Capsium annuum* after boiling.

### Antimicrobial activity

**Figure 7:**
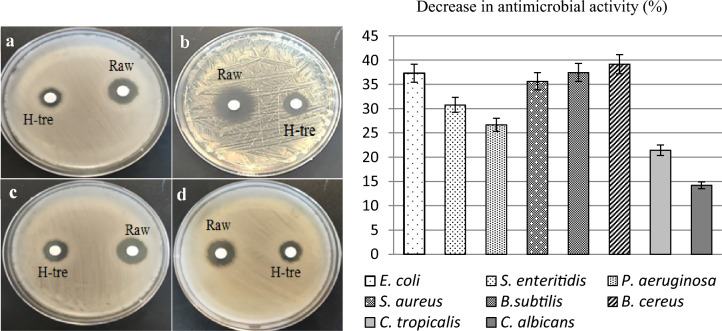
Antimicrobial activities of raw and heat-treated extracts against *P. aeruginosa* (**a**), *S. aureus* (**b**), *C.albicans* (**c**), *C. tropicalis* (**d**) and the decrease in antimicrobial activity. H-tre: heat-treated.

Antimicrobial activities of raw and heat-treated *O. umbellatum* extracts are given in Table 3. Different degrees of inhibition were obtained against all tested microorganism strains. The highest activity of raw extract was determined against *S. aureus* as gram-positive bacteria with an inhibition zone of 21.9 ± 0.7 mm, and against *P. aeruginosa* as gram-negative bacteria with an inhibition zone of 16.5 ± 0.3 mm. The fact that the extract forms an inhibition zone on all bacteria indicates that it has a broad spectrum. However, it was also determined that the *O. umbellatum* extract exhibited higher activity against gram-positives compared to gram-negatives. This selectivity against bacteria is due to the structural difference between gram-positive and gram-negative bacteria. The double membrane surrounding the cell in Gram-negative bacteria is the main cause of resistance to antimicrobial compounds. While all bacteria have an inner membrane, gram-negative bacteria have a selective outer membrane. This outer membrane provides resistance to cidal effects by preventing many compounds from entering the cell. Since this selective outer membrane is absent in Gram-positive bacteria, antimicrobial compounds show higher activity in these bacteria. In addition, the cell wall of gram-positive bacteria has a highly porous structure, while the cell wall of gram-negative bacteria has a high lipid content and the penetration of antimicrobials into the cell depends on the outer membrane proteins^40^. *O. umbellatum* extract has affected fungal species as much as gram-positive and gram-negative bacteria. Inhibition zones of 15.4 ± 0.5 mm and 16.2 ± 0.3 mm against *C. tropicalis* and *C. albicans*, respectively, were obtained with the raw extract. The maximum inhibition zones obtained with the raw extract against gram-positive (*S. aureus*), gram-negative (*P. aeruginosa)* and fungi are given in Fig. 7. The broad-spectrum effect of the raw extract comparable to standard antibiotics can be explained by the active ingredients. Phenolic compounds detected by LC–MS/MS analysis have an important effect on the emergence of antimicrobial activity. Procateuic acid, which is determined as the major compound in the content, has an inhibitory effect on the proliferation of bacteria and strengthens the inhibition by showing a synergistic effect with many antimicrobial compounds^41^. The high level of gallic acid detected in the extract contributes to the antimicrobial activity of the extract by inhibiting the protein flow pumps in bacterial membranes, arginase activity and folate synthesis^42^. Both the minor and major components of the extract enhance antimicrobial effect. Vanillin, which is detected in minor amounts in the extract, disrupts the integrity of the bacterial membrane, causes ions in the cell to leak out, and prevents intracellular pH homeostasis^43^. A strong antimicrobial activity emerges as a result of the cumulative effect of all active ingredients detected in the extract. The antimicrobial activity of *O. umbellatum* grown in different ecological environments has also been reported by some literature studies. Demirkol et al.^9^ reported that extracts of the *O. umbellatum*, which is distributed in Ordu (Turkiye), exhibited a broad-spectrum activity, but did not show any activity against *Listeria monocytogenes*. Yiğit et al.^10^ reported that *O. umbellatum* collected from Erzincan (Turkiye) did not show any effect against *P.aeruginosa*, *E. coli, E. Aerogenes* and *S. aureus*. These differences reported in the literature studies can be explained by the differences in the secondary metabolites and their levels produced during the adaptation process of the plant to different ecological conditions.

**Table 3:** Inhibition zones (mm) of raw and heat-treated *O. umbellatum* extract.

	Microorganisms	Raw	Heat-treated	Amikacin	Nistatine
Gram negative	*E. coli*	12.6 ± 0.4	7.90 ± 0.1	17.9 ± 0.7	Nt
	*S. enteritidis*	11.7 ± 0.3	8.10 ± 0.2	16.5 ± 0.4	Nt
	*P. aeruginosa*	16.5 ± 0.3	12.1 ± 0.4	17.8 ± 0.5	Nt
Gram positive	*S. aureus*	21.9 ± 0.7	14.1 ± 0.6	22.5 ± 0.8	Nt
	*B.subtilis*	18.7 ± 0.4	11.7 ± 0.5	21.1 ± 0.6	Nt
	*B. cereus*	17.1 ± 0.9	10.4 ± 0.7	18.3 ± 0.2	Nt
Fungi	*C. tropicalis*	15.4 ± 0.5	12.1 ± 0.4	Nt	16.9 ± 0.4
	*C. albicans*	16.2 ± 0.3	13.9 ± 0.2	Nt	17.8 ± 0.2

Nistatine 30 µg/mL, Amikacin 30 μg/mL, Nt: not tried.

The heat treatment applied during cooking causes changes in the phytochemical content of foods. In LC–MS/MS, ICP-MS and FTIR analyses, it was determined that the heat treatment application caused changes in the phytochemical content of *O. umbellatum*. Along with these changes, the application of heat caused a change in the antimicrobial activity of *O. umbellatum*. In *S. aureus*, where the highest zone of inhibition was observed, the raw extract formed a zone of inhibition of 21.9 mm, whereas the heat-treated extract was able to form a zone of 14.1 mm. Heat treatment caused a decrease in the antimicrobial activities against bacteria at varying rates. The most significant reduction in antimicrobial activity among all tested bacteria was obtained against *B. cereus*, and heat treatment decreased the antimicrobial activity of the raw extract against *B. cereus* by 39.2%. The reductions in antimicrobial activity determined against all tested microorganisms are given in Fig. 7. The decreases in antimicrobial activities are directly related to the decrease in phytochemical content. Changes in the content of procateuic acid and gallic acid, which are major components and have antimicrobial activity, are the biggest reasons for the decrease in antimicrobial activity. While procateuic acid was found at the rate of 53.136 mg/kg in the raw extract, its amount was determined as 5.29 mg/kg after heat treatment. A similar decrease was also detected in gallic acid, and its rate which was 41.722 mg/kg decreased to 3.046 mg/kg after heat treatment. Similar decreases were detected in all phenolic compounds detected in phytochemical content, and these decreases directly affected the antimicrobial activity. Although there is no study in the literature investigating the effect of heat treatment on the phytochemical content and antimicrobial activity of *O. umbellatum*, it is reported that the activity of many extracts decreases with the effect of heat. Ginovyan^44^ observed that *Hypericum alpestre* extract lost its antimicrobial activity after 60 min of heat treatment at 60 °C. Sah et al.^45^ reported that the zone of inhibition developed by *Allium sativum* extract against *K. pneumoniae* decreased from 26 to 17 mm after heat application at 100 °C, and that *Zingiber officinale* extract, which exhibited a 20 mm zone, completely lost its antimicrobial activity.

### Antimutagenic activity

**Figure 8:**
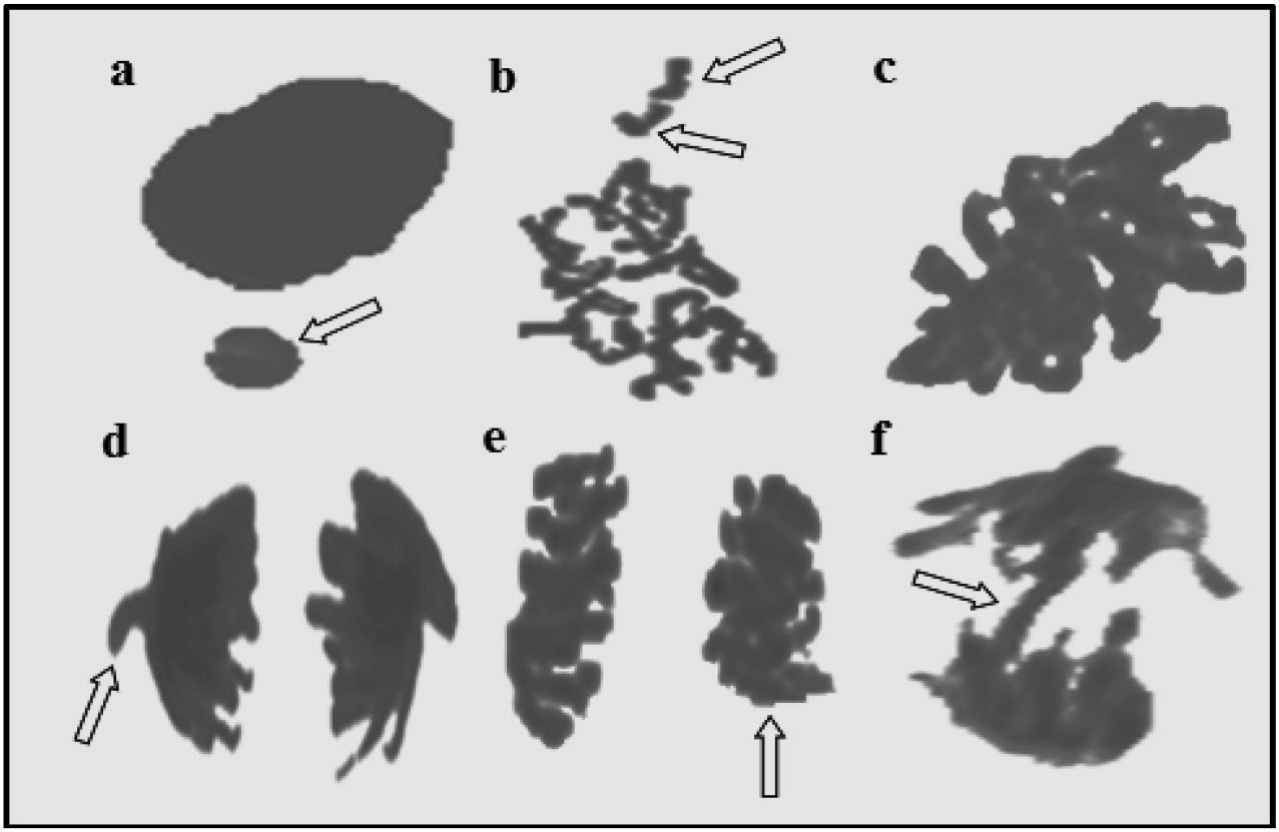
MN and CAs induced by NaN_3_. MN (**a**), fragment (**b**), sticky chromosome (**c**), vagrant chromosome (**d**), unequal distribution of chromatin (**e**), bridge (**f**).

The antimutagenic activities of raw and heat-treated *O. umbellatum* extracts are given in Table 4. While low levels of MN and CAs formations were observed in the control group and only the extract-treated groups (p > 0.05), high levels of fragments, sticky chromosome, unequal chromatin distribution, bridge and MN formations were observed in the NaN_3_-treated group. Among the CAs, the highest level of fragment abnormalities was found (Fig. 8). NaN_3_ is a chemical mutagen and its mutagenicity occurs through the production of an organic metabolite of the azide compound. This metabolite enters the nucleus, interacts with DNA, causing mutations and instabilities in the genome^46^. The antimutagenic activities of the extracts were determined by considering the reduction in the frequencies of MN and CAs induced by NaN_3_. The raw extract caused regression of all NaN_3_-induced abnormalities by more than 50%, except for the vagrant chromosome. Raw extract, which provided 43.2% regression in the vagrant chromosome, showed the strongest antimutagenic effect as 69.8% in the unequal distribution of chromatin. Gallic acid, which is intensely detected in the extract, exhibits antimutagenic activity by clearing electrophilic mutagens and binding to outer membrane carriers and preventing the transfer of mutagens to the cytosol^47^. Caffeic acid detected in the extract content exhibited an antimutagenic effect through antioxidative activity by preventing the formation of hydroxyl radicals and suppressing free radical production^48^. Similarly, protocatechuic acid has important antimutagenic properties. Anter et al.^49^ reported that protocatechuic acid has antigenotoxic potential in in-vitro studies, Olvera-Garcia et al.^50^ reported that protocatechuic acid exhibited antimutagenic activity by inhibiting the mutagenicity of nitropyrene.

**Table 4:** Effect of raw and heat-treated extracts on the frequency of MN and CAs induced by NaN_3_.

	Negative control	Positive control (NaN_3)_	Raw extract	Heat-treated extract	Raw extract + NaN_3_	Heat-treated extract + NaN_3_
MN	0.20 ± 0.42^d^	82.80 ± 8.66^a^	0.00 ± 0.00^d^	0.20 ± 0.42^d^	33.40 ± 8.07^c^	69.50 ± 6.54^b^
FRG	0.31 ± 0.17^d^	83.50 ± 10.68^a^	0.00 ± 0.00^d^	0.32 ± 0.16^d^	31.60 ± 6.45^b^	56.30 ± 6.17^c^
SC	0.00 ± 0.00^d^	62.80 ± 8.13^a^	0.41 ± 0.13^d^	0.00 ± 0.00^d^	26.00 ± 6.31^c^	47.90 ± 6.59^b^
VC	0.00 ± 0.00^d^	51.80 ± 9.87^a^	0.55 ± 0.19^d^	0.00 ± 0.00^d^	29.40 ± 7.37^c^	42.30 ± 6.65^b^
UDC	0.00 ± 0.00^d^	38.20 ± 9.90^a^	0.00 ± 0.00^d^	0.74 ± 0.21^d^	11.50 ± 6.62^c^	29.70 ± 6.62^b^
B	0.00 ± 0.00^d^	26.70 ± 8.55^a^	0.00 ± 0.00^d^	0.00 ± 0.00^d^	11.70 ± 3.59^c^	26.5 ± 6.22^b^

MN: micronucleus, FRG: fragment, SC: sticky chromosome, VC: vagrant chromosome, UDC: unequal distribution of chromatin, B: bridge. 1.000 cells were counted in each group for MN and CAs.

Means shown with different letters^(a-d)^ on the same line are statistically significant (p < 0.05).

**Figure 9:**
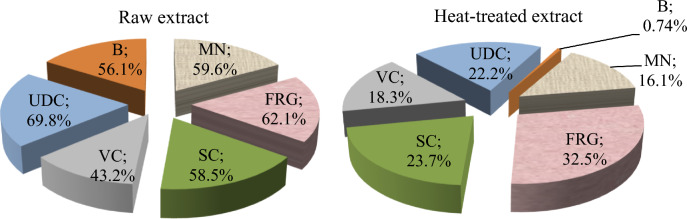
Antimutagenic activities of raw and heat-treated extracts. MN: micronucleus, FRG: fragment, SC: sticky chromosome, VC: vagrant chromosome, UDC: unequal distribution of chromatin, B: bridge.

Heat treatment caused significant changes in antimutagenic activity and in general, significant decreases were observed in the antimutagenic activity exhibited by the raw extract against all abnormalities. The raw extract exhibited antimutagenic activity by reducing bridge by 56.1%, while heat-treated extract reduced bridge frequency by 0.74%. Briefly, the heat treatment caused the loss of the antimutagenic activity of the raw extract against bridge formation. Significant reductions in antimutagenic activity against other abnormalities were also observed after heat application (Fig. 9). Heat treatment decreased the antimutagenic activity of the raw extract against unequal distribution of chromatin by 68.2%, and the antimutagenic activity against MN formations by 73.1%. The reducing effect of heat treatment on antimutagenic activity is directly related to decreases in phytochemical content. The decreases in the amount of major compounds such as gallic acid, protocatechuic acid and caffeic acid detected in the content were also reflected in the antimutagenic activity. Although there is no study in the literature investigating the effect of heat treatment on the antimutagenic activity of *O. umbellatum*, it is reported that the activity of many extracts decreases with the effect of heat. Wang et al.^51^ reported that the antimutagenic activity of *Hericium erinaceus* extract decreased after heat treatment and the antimutagenic activity was heat sensitive.

### Antiproliferative activity

The antiproliferative activities of raw and heat-treated *O. umbellatum* extracts are given in Table 5. A total of 5.000 cells were counted in each group and the cells in prophase, metaphase, anaphase, telophase were considered as dividing cells. While a total of 245 cells in the negative control group were in the dividing stage, this number decreased to 110 with the administration of glyphosate. Glyphosate is a compound that inhibits cell division and acts at the G_2_/M transition point by causing abnormalities in cell cycle checkpoints^52,53^. Because of these effects on the cell cycle, it is used as a positive control in antiproliferative studies. It was determined that raw and heat-treated *O. umbellatum* extracts decreased cell proliferation compared to the negative control group. Raw extract reduced the number of dividing cells from 245 to 196 and reduced the cell proliferation by 20%. This decrease in cell proliferation indicates the antiproliferative effect of *O. umbellatum* extracts. Many phenolic compounds have a reducing effect on cell proliferation^54,55^. Gallic acid, which is concentrated in the raw extract, is an important compound that exhibits increasing antiproliferative activity in a dose-dependent manner^56^. Caffeic acid, on the other hand, exhibits antiproliferative activity by causing changes in mitochondrial membrane potential^57^. As a result of the cumulative effect of the phenolic compounds in the extract, the raw extract exhibits antiproliferative activity. Although there is no study investigating the antiproliferative activity of *O. umbellatum* extract in the literature, there are studies reporting the effect of many natural plant extracts on cell proliferation. Frescura et al.^58^ reported that the leaf and bark extracts of *Luehea divaricata* exhibit antiproliferative effects in *A. cepa*, i.e., it has activity that can inhibit cell division. Cragg and Newman^59^ reported that many of the agents used in cancer treatment were obtained from natural sources and were discovered in cell proliferation inhibition tests.

**Table 5:** Anti-proliferative effects of raw and heat-treated extracts.

	Dividing cell number^a^	Number of cells in interphase	MI (%)
Negative control	245	4755	4.90
Positive control (glyphosate)	110	4890	2.2
Raw extract	196	4804	3.92
Heat-treated extract	226	4774	4.52

^a^İndicates the number of cells in prophase, metaphase, anaphase and telophase.

After heat application, a decrease in the antiproliferative activity of the *O. umbellatum* extract was observed, and the heat-treated extract caused a 7.6% regression in cell proliferation compared to the negative control. This decrease in antiproliferative activity is directly related to the decrease in phenolic content. In literature studies, Ferrarini et al.^60^ reported that the heat treatment during microwave, boiling and baking processes decreased the antiproliferative activity of Brussels sprouts. Agents that reduce MI by 22% have lethal effect compared to negative control, while agents that reduce 50% usually have sublethal effects^20^. In this study, it was determined that both the raw and heat-treated extract exhibited antiproliferative activity at a level far from the lethal/sublethal limits and was not cytotoxic compared to the positive control.

## Conclusion

Plants are natural foods with many biological and pharmacological activities. Many plant species used as food are consumed by various cooking methods. Heat treatment of foods results in biological, physical, and textural changes, differences in phytochemical content, and reduction or increase in biological activity. Although there are many studies investigating the phytochemical content and biological activity of herbal extracts, the level of studies investigating the changes in phytochemical content and biological activity of the extracts with the cooking process is not at the desired level. The effect of heat should not be ignored, especially in studies carried out with plants consumed by cooking. In this study, the effect of heat treatment on the phytochemical content and biological activities of *O. umbellatum* was investigated. As a result, both the raw and heat-treated extract had antioxidant, antimicrobial, antimutagenic and antiproliferative effects without exhibiting cytotoxic and mutagenic effects. After heat treatment some anti-nutritional factors (such as Cd, As, V) were inactivated and there was a significant decrease in phenolic compounds of *O. umbellatum*. The decrease in the active ingredient content of *O. umbellatum* after heat treatment may be related to the decomposition of compounds and extraction by water. The passage of various macro and micro molecules into the boiling water enriches the water in terms of phytochemicals and makes it valuable. For this reason, it is very important to evaluate the boiling water produced during cooking. Heat treatment also caused a decrease in antioxidant, antimicrobial, antimutagenic and antiproliferative activities along with a decrease in phenolic content. Since plant foods are indispensable parts of our daily diet, the gentlest cooking methods to preserve phytochemicals and their mechanisms of action on phytochemicals should be investigated.

### Supplementary Information

Supplementary Information.

## Data Availability

The datasets used and/or analyzed during the current study are available from the corresponding author on reasonable request.
