# Supplementary material for: Phytochemical fingerprint and biological activity of raw and heat-treated Ornithogalum umbellatum
Source: Sci Rep. 2023 Aug 23;13:13733. doi: 10.1038/s41598-023-41057-w (PMC10447479; doi:10.1038/s41598-023-41057-w)
Supplement: Supplementary file 1 — Supplementary Information. [file 41598_2023_41057_MOESM1_ESM.docx]

Total chromatogram of standart compounds

b

a

c

d

e

f

Chromatogram of standart compounds a. gallic acid, b. procateuic acid, c. procateuic aldehyde, d. catechin and epicatechin, e. caffeic acid, f. vanillin

a

b

c

d

f

e

Chromatogram of standart compounds a. taxifolin, b. p-coumaric acid, c. ferulic acid, d. rosmarinic acid, e. oleuropein, f. OH-benzoic acid

f

e

d

c

b

a

Chromatogram of standart compounds a. salicylic acid, b. rutin, c. resveratrol, d. ellagic acid, e. quercetin, f. kaempferol
